# Preparation and Load-Bearing Capacity of Lattice Cell Warren Truss Slot Resin-Stiffener-Reinforced Foam Sandwich Material

**DOI:** 10.3390/ma16072729

**Published:** 2023-03-29

**Authors:** Xueshan Chen, Wei Tian, Xiaoke Jin, Chenyan Zhu

**Affiliations:** 1 College of Textile Science and Engineering, International Institute of Silk, Zhejiang Science & Technology University, Hangzhou 310018, China; 2 College of Material and Textile Engineering, Jiaxing University, Jiaxing 314001, China

**Keywords:** warren truss slot-hole structure, foam sandwich composite, flat compression, bending, load, load-bearing capacity, lattice cell, resin-reinforced stiffener

## Abstract

This study optimized and proposed a Warren truss slot-hole structure with a double-sided, square shallow slot and vertical and horizontal corrugated symmetry, achieved with inclined holes based on the stability and a good bearing capacity of an inclined strut truss structure. The tetrahedral truss lattice cells were obverse and reverse-staggered in the central core of the structure. Compared with the double-sided, square shallow groove cylindrical straight hole, the resin consumption of the Warren truss slot holes was similar to that of a vacuum-assisted resin infusion; however, the external flat compression force of the Warren truss slot holes on the resin stiffener structure doubled, and its bending contact force increased by approximately 1.5 times. Furthermore, the resulting Warren truss-slotted resin structure exhibited a late failure time. Compared with the double-sided, square shallow groove cylindrical straight hole foam-core sandwich composite, the Warren truss slot resin-stiffener-reinforced sandwich composite exhibited an increase of 4.7 kN in the flat compression load, an improvement of ~40% in flat compressive strength performance, an increase of ~0.58 kN in the bending load, and an improvement of ~60% in the bending strength, demonstrating its better bearing strength performance.

## 1. Introduction

Foam-core sandwich composites have been garnering popularity in the research community owing to their high-specific stiffness, specific strength, low density, and lightweight characteristics [1,2,3]. The applications of sandwich composites have evolved rapidly, with extensive use in aerospace, rail transportation, wind power blades, naval structure design, and other fields [4,5,6]. In the vacuum-assisted resin infusion (VARI) molding process of sandwich composites, the foam-core grooves and internal holes improve the infiltration of the fiber fabric of the upper and lower composite skins [7], thereby increasing the bond strength between the structural layers and enhancing the strength of the sandwich composite material. However, as the foam-core grooves and holes are manufactured by semi-automatic XYZ triaxial cutting and drilling, the slot structure consists of shallow grooves and circular through-holes. Thus, the reinforcement structure formed after resin infiltration and curing remains unstable; this instability can cause rapid structural failure when the structure is subjected to external loads (compression or bending) and consequently reduces the reinforcing effect [8,9,10]. Therefore, foam-core slot structures and sandwich composite reinforcement should be enhanced to achieve a high and stable load-bearing capacity without increasing the amount of injected resin.

To improve the mechanical properties of sandwich composites, fiber filling, stitching, Z-pin reinforcement, and other methods have been thoroughly investigated. Liu et al. [11] designed a novel, glass-fiber-reinforced, concrete lattice sandwich panel by filling the foam cubes with orthogonal corrugated truss fibers, followed by a resin injection. Sayahlatifi et al. [12] combined fiber fabric and corrugated balsa wood into a corrugated hybrid core, thereby substantially improving the specific strength and stiffness of the hybrid core sandwich composite. Li et al. [13] prepared a sandwich composite with an integrated orthogrid stiffened syntactic foam core by filling the syntactic foam in the bays or cells of the fiber-reinforced polymer orthogrid skeleton, thereby enhancing its energy transfer and absorption impact. Xu et al. [14,15] presented a sandwich material by stitching the upper and lower skin layers to the core layer to improve the flexural and climbing drum peel properties. Wei et al. [16] used integrated stitching to prepare sandwich composites and demonstrated that the flatwise compression strength and compression modulus were proportional to the stitching yarn volume fraction. Chou et al. [17] prepared stitch-reinforced sandwich panels with various stitch angles, whereby smaller stitching angles were suggested to improve the flexural properties of the material. Nanayakkara et al. [18] improved the compression modulus, compression strength, and energy absorption of sandwich composite materials by Z-pin reinforcement. Tao et al. [19,20] inserted additional fiber yarns into the grooves of the foam core to form a fiber-reinforced resin composite stiffener to improve the bond strength of the core with the skin layer and Z-directional mechanical properties of the sandwich composite. Kay et al. [21] improved the fatigue and mechanical properties of polyurethane foam-core sandwich composites by using a three-dimensional (3D) continuous fiber reinforcement scheme. Based on previous studies, fiber filling, stitching, Z-pin reinforcement, and stiffener insertion can improve the mechanical properties of foam-core sandwich composites. However, these methods rely heavily on stitching, Z-pinning equipment, or a manual layup, which reduces the vacuum infusion molding speed during the sandwich composite preparation, thereby hindering the large-scale application of sandwich composite VARI molding in wind power or boat manufacturing. 

To address these problems, researchers utilized the VARI molding process to enhance the sandwich composite performance with a foam-core-grooved resin stiffener. Yokozeki et al. [22] improved the debonding toughness of the foam-core sandwich panels by core cutting. Nejad et al. [23] prepared through-thickness polymers and pin-reinforced sandwich panels using foam cores with through-thickness holes to enhance their flexural properties. Jayaram et al. [24] produced polyester pin-reinforced sandwich panels using a through-thickness honeycomb foam panel as the core layer, which effectively enhanced the interfacial strength between the face and core layer and improved the overall structural properties of the sandwich panels. 

The foam-core panels in the aforementioned studies were processed using semiautomatic triaxial cutting and drilling, and the slot resin reinforcement structure was formed by infiltrating the resin into the core material, followed by curing. Although the reinforcement effect on the sandwich composites was analyzed extensively, limited research has been conducted on slot structure optimization and achieving excellent mechanical properties in the cases of the trusses and lattice structures, and improving the bearing capacity of the slotted resin reinforcement. In addition, the stability and the bearing capacity further improved the slot-hole resin-reinforced stiffener structure; thus, by taking advantage of the excellent mechanical performance of the truss and lattice, the slot-hole structure can be optimized further and the bearing capacity of the foam-core sandwich composite can be improved. However, these have been thoroughly studied. In particular, the nature of the semiautomatic triaxial foam-core panel processing may produce non-perpendicular, straight, cylindrical holes and square shallow surface grooves, thus resulting in an unstable resin reinforcement structure. When stressed, these resin reinforcement structures are prone to brittle fracture owing to the bending moment. 

**Figure 1:**
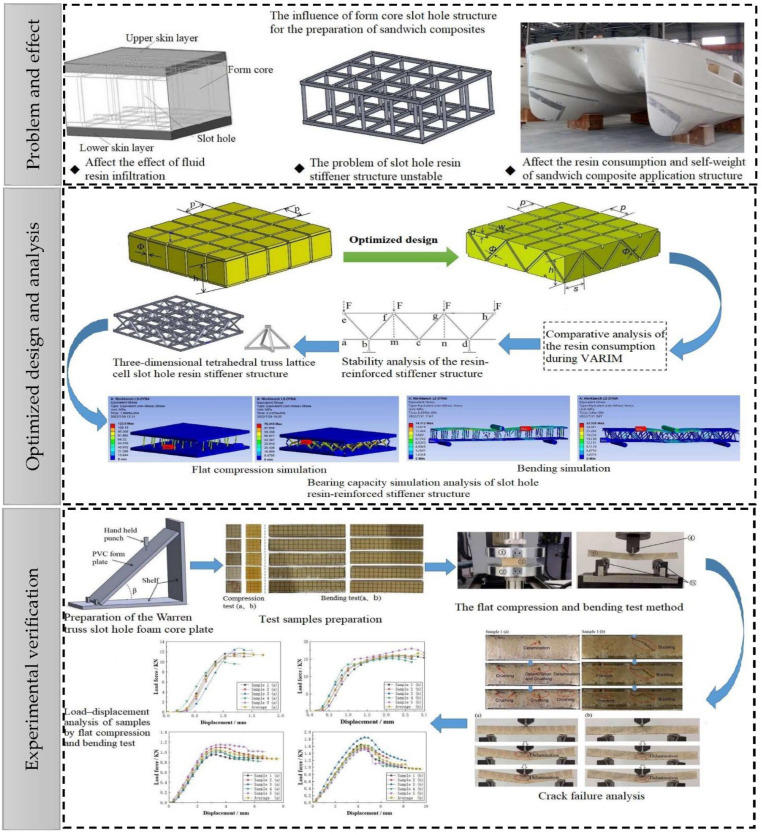
Technical flowchart for the comprehensive characterization of the improvement of the foam-core sandwich composite’s capacity and strengths from the problem analysis of the slot-hole structure, which is optimized to a Warren truss slot-hole structure, and the advantages of the optimized slotted resin-stiffener-reinforced sandwich composites are verified by experiment.

This study describes the stability of an inclined strut truss structure and strong bearing capacity of the lattice cell. A double-sided, square shallow groove, and cylindrical through-hole structure were transformed into a Warren truss slot-hole structure, which was composed of double-sided, square shallow slots, and vertically and horizontally corrugated symmetrically inclined through-holes. The central core structure comprised tetrahedral truss lattice cells with obverse- and reverse-staggered arrangements. The bearing capacity of the slot resin reinforcement structure was investigated, and experiments were designed to evaluate the enhancement effect of the slot resin reinforcement on the flat compression and bending strengths of the foam-core sandwich composites. The flowchart summarizing all the procedural steps executed to assess stability is shown in Figure 1. It is of great significance to further improve the mechanical properties of foam-core sandwich composite structural materials with fiber-fabric-reinforced skin layers in order to enhance the performance of the key structural components of the watercraft, such as the bottom frame and bulkhead. 

## 2. Analysis of the Influence of a Foam-Core Slot-Hole Structure on the Preparation of the Sandwich Composite Using VARIM

### 2.1. Analysis of the Influences of the Foam-Core Slot-Hole Structure

#### 2.1.1. Effects of the Slot-Hole Structure on VARIM

**Figure 2:**
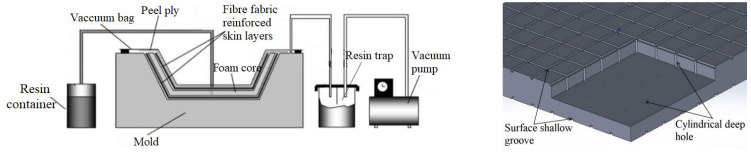
VARIM of the sandwich composites and its form core.

In the process of manufacturing sandwich composites using VARIM, at a high-strength negative pressure, the resin fluid simultaneously infiltrates the reinforced fiber fabric of the upper skin layer and the grooves and holes on the upper surface of the foam core. From the top to bottom parts of the structure, the fluid flows through the holes, and infiltrates the grooves on the lower surface of the foam core and reinforced fiber fabric of the lower skin layer, as shown in Figure 2. The viscosity of the fluid resin gradually increased and the resin was cured.

#### 2.1.2. Effects of the Slot-Hole Structure on the Resin Consumption in VARIM and Self-Weight of the Sandwich Composites

In the manufacturing of large sandwich composite structures by VARIM, the amount of resin was strictly calculated and controlled. For example, a composite structure could be constructed using a 1 m × 1 m, 5.94 kg sandwich-composite panel with a weight of 5.94 kg and a density of 60 kg·m^−3^; this panel contained 25 mm double-sided, square shallow grooves, and straight cylindrical holes, and foam core was used as the sandwich layer; 0°90°LT800 glass fiber biaxial cloth was used in the upper and lower skin layers; and unsaturated polyester (ρ: 1100 kg·m^−3^) was used as the resin matrix. The weight ratios of these materials are outlined in Table 1.

As shown in Table 1, the injected core slot structure resin accounts for 15.3% of the total weight of the sandwich composite panel and approximately 46.7% of the total resin consumption. Therefore, in the manufacturing of large sandwich composite structures, the foam-core slot-hole structure is an important factor that determines the consumption of the resin and the self-weight of the sandwich composite.

#### 2.1.3. Effect of the Slot-Hole Resin-Reinforced Stiffener Structure

**Figure 3:**
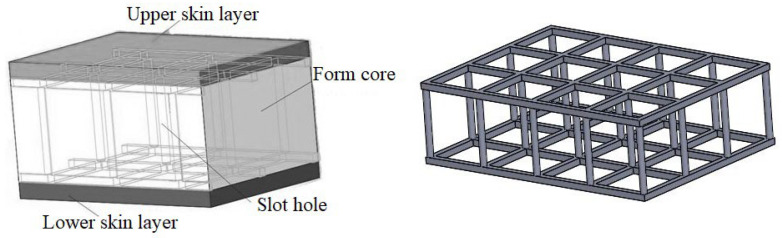
Diagram of the slot resin-reinforced sandwich composite and the resin stiffener structure.

During the VARI molding process, resin was injected into the slots, and the resin in the grooves on the lower and upper surface of the foam core and in the holes inside of the form core cured and formed a resin-reinforced stiffener. The slot-hole resin-reinforced stiffener structure is helpful to improve the bonding property between the skin layer and the core layer, and it enhances the bearing strength of the sandwich composite. The slot resin structure can be regarded as an independent reinforcement structure within the sandwich material. A resin-reinforced structure with double-sided square shallow grooves and straight cylindrical holes is illustrated in Figure 3.

### 2.2. Stability Analysis of Resin-Reinforced Stiffener with Square, Double-Sided Shallow Grooves, and Cylindrical Straight Holes

#### 2.2.1. Analysis of the Plane Structure of Resin-Reinforced Stiffener Structure

**Figure 4:**
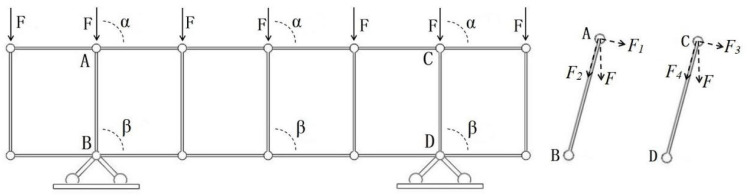
Force diagrams of the plane truss structure and two web members.

The triaxially processed foam panel slot consisted of shallow grooves on the surface and straight cylindrical through-holes. The resin cured in the grooves and holes and formed the resin-reinforced stiffener; its shape characteristics are among the key factors that affected the bearing capacity and overall reinforcement effects. The planar structure of the resin reinforcement with the square, double-sided shallow grooves, and cylindrical straight holes can be described as a parallel chord plane-truss structure. In particular, the shallow grooves in the upper layer constitute the top chord, the cylindrical straight holes constitute the web, and the lower surface shallow grooves constitute the chord at the bottom part. A force diagram of the plane truss structure is shown in Figure 4.

Figure 4 shows an ideal truss structure without a tilt-rod support. This structure is an ideal steady-state truss when the angles between action F and the top chord (α), and between the web, top, and lower chords (β) are exactly 90°. Web AB and CD were subjected to axial forces of equal magnitude in opposite directions. When α or β are not 90°, the structure becomes unstable, and can no longer be described as a truss. In addition to the axial force *F*_2_, web AB is subjected to the torque generated by force *F*_1_ (M = *L* × *F*1), as shown in Figure 2, where *L* is the web length, *F*_1_ is the force component perpendicular to the web, and *F*_2_ is the axial force component.

#### 2.2.2. Analysis of the 3D Structure of Resin-Reinforced Stiffener Structure

**Figure 5:**
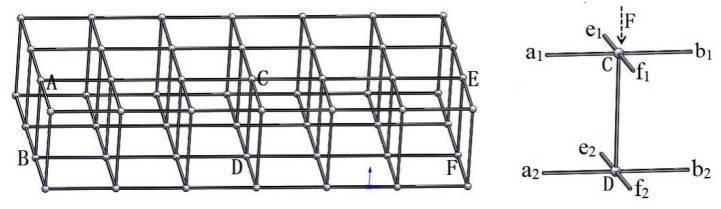
Three-dimensional truss structure and force diagram of its web member.

The space truss and local force analysis of the resin reinforcement structure with double-sided square shallow grooves and cylindrical straight holes are shown in Figure 5. An ideal and 3D truss structure is obtained when the direction of force F is exactly perpendicular to the transverse top chord a_1_b_1_ and longitudinal top chord e_1_f_1_, and when the web CD is perpendicular to the bottom chords a_2_b_2_ and e_2_f_2_. Without a tilt-rod support, the force F acting on the truss is not along the direction of the web; hence, F is not perpendicular to the top and bottom chords, which results in the web rod being subjected to the axial force and torque generated by the perpendicular force component. Consequently, the sample undergoes transverse or longitudinal torsional deformation, leading to a web fracture within the cylindrical straight hole, which weakens the overall bearing capacity of the resin reinforcement.

The foam-core slot structure was processed by semiautomatic triaxial cutting and drilling. Owing to the manual placement, cutting and drilling errors, and other factors, the fabricated cylindrical straight hole and the transverse and longitudinal shallow grooves on the surface were not perpendicular to each other; this resulted in an unstable resin-reinforced slot structure. When subjected to an external force, the resin-reinforced web becomes susceptible to brittle fracture owing to the bending moment, which affects the overall load-bearing capacity of the resin-reinforced structure and the strength of the sandwich composite.

## 3. Optimized Design and Analysis of the Foam-Core Slot-Hole Structure 

### 3.1. Optimized Design of the Foam-Core Slot-Hole Structure

**Figure 6:**
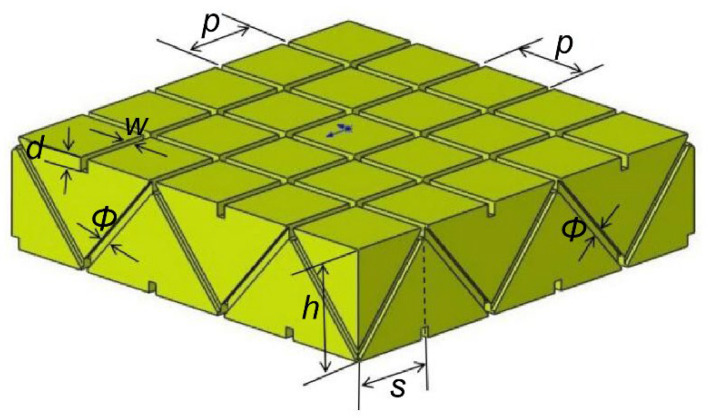
Warren truss slot structure with double-sided square shallow grooves and symmetrical inclined holes. *p* is the groove spacing, *w* is the shallow groove width, *d* is the groove depth, *Φ* is the symmetrically inclined hole diameter, *s* is the inclined span of the hole, and *h* is the inclined hole height.

Trusses are extensively used in large-scale structural engineering because of their light weight, increased bearing capacity and diverse structural designs. An inclined rod support is necessary to stabilize the truss. Section 2.1.1 discussed the instability of the resin-reinforced structure with double-sided square shallow grooves and cylindrical straight holes without an inclined rod support. Thus, this study optimized the existing resin reinforcement structure into a Warren truss with double-sided square shallow grooves and symmetrically inclined holes, as illustrated in Figure 6. The dimensions of the structural parameters are presented in Table 2.

### 3.2. Structural Analysis

#### 3.2.1. Comparative Analysis of Resin Consumption During VARIM

During the VARI molding of the sandwich composites, resin was primarily injected into the grooves and holes of the core material and it infiltrated the fabric of the upper and lower skin layers. The groove and hole sizes are key factors to determining the resin consumption and manufacturing costs. In this study, the slot structure was composed of cylindrical straight or symmetrically inclined holes, and the hole center was placed at the intersection of the longitudinal and transverse squared shallow grooves. Given that the unit structure, shape, size, and other parameters are fixed, the volume of the foam-core slot can be estimated with various areas to determine the required amount of resin for production. The volume of the double-sided, square shallow groove, and cylindrical straight hole can be expressed as,

(1) $$V=\;N_{1}V_{1}+M_{1}V_{2},$$

(2) $$\begin{array}{ll} =2\times \left(\frac{S}{X^{2}}\right)\times & \left\{\left(X\times \left(\frac{W_{1}}{2}\right)\times D_{1}\right)\times 2+\left[\left(X–W_{1}\right)\times \left(\frac{W_{1}}{2}\right)\times D_{1}\right]\times 2\right\}+\\ & \left(\frac{S}{X^{2}}\right)\times \left[4\times \left(T\;–\;2D_{1}\right)\times \pi \times {\left(\frac{\Phi }{2}\right)}^{2}\times \left(\frac{1}{4}\right)\right], \end{array}$$

where *X* is the surface groove spacing and center-to-center spacing of the cylindrical straight holes (mm), *S* is the area of the foam-core panel (mm^2^), *N_1_* is the number of straight cylindrical holes, *M*_1_ is the number of grooves, *V*_1_ is the volume of the cylindrical hole (mm^3^), *V*_2_ is the volume of the shallow groove (mm^3^), *W*_1_ is the width of the shallow groove, *D*_1_ is the depth of the groove (mm), *Φ* is the diameter of the cylindrical hole (mm), and *T* is the thickness of foam-core panel equal to 25 mm. According to Equation (2), a foam-core panel with a thickness of 25 mm and a volume of 924.93 cm^3^ for the double-sided square shallow grooves and cylindrical straight holes has a resin consumption equal to 924.93 mL.

The volume of the Warren structure with longitudinal and transverse grooves and symmetrical inclined holes can be expressed as

(3) $$V=N_{2}V_{1}+M_{2}V_{2},$$

(4) $$=\left(\frac{S}{X^{2}}\right)\times V_{1}+\left(\frac{S}{X^{2}}\right)\times V_{2},\;$$

(5) $$\begin{array}{ll} =\{\left(X\times \left(\frac{W_{1}}{2}\right)\times D_{1}\right)\times 2 & +\left[\left(X\;–W_{1}\right)\times \left(\frac{W_{1}}{2}\right)\times D_{1}\right]\times 2\}\times 2+\left(\frac{S}{X^{2}}\right)\times 4\times \left(\frac{1}{4}\right)\times \\ & \pi {\left(\frac{\Phi }{2}\right)}^{2}\times \sqrt{{\left(T-2D_{1}\right)}^{2}+\left(\frac{X^{2}}{4}\right)}, \end{array}$$

where *X* is the shallow groove spacing on the surface (mm), *S* is the area of the foam-core panel (mm^2^), $N_{2}$ = the number of double-sided square shallow grooves within a given area, *V*_1_ is the volume of the grooves (mm^3^), *M*_2_ is the number of inclined cylindrical holes within a single square of the shallow groove, *V*_2_ is the volume of the inclined cylinders (mm^3^), *W*_1_ is the width of the shallow grooves (mm), *D*_1_ is the depth of the shallow grooves (mm), *Φ* is the diameter of the inclined cylindrical holes (mm), and *T* is the thickness of the foam-core panel equal to 25 mm.

According to Equation (5), a foam-core panel with a thickness of 25 mm and a volume of 942.68 cm^3^ for the Warren truss slot structure with double-sided shallow grooves and symmetrical inclined holes has a resin consumption of 942.68 mL. Compared with the structure with double-sided square shallow grooves and cylindrical straight holes, the amount of resin required to infiltrate the grooves of a 1 m^2^ Warren truss slot foam board increased by 17.75 mL, which can incur little to no additional manufacturing costs. 

Therefore, when the original foam-core panel slotted-hole structure design is optimized into a stable Warren truss slot structure, the symmetrical diagonal resin web reinforcement is axially stressed, regardless of whether the external force acts vertically on the upper chord of the slotted resin reinforcement structure, and the structure becomes more stable. The resin consumption of the novel design was approximately the same as that of the original structure. 

#### 3.2.2. Stability Analysis of the Resin-Reinforced Stiffener Structure

**Figure 7:**
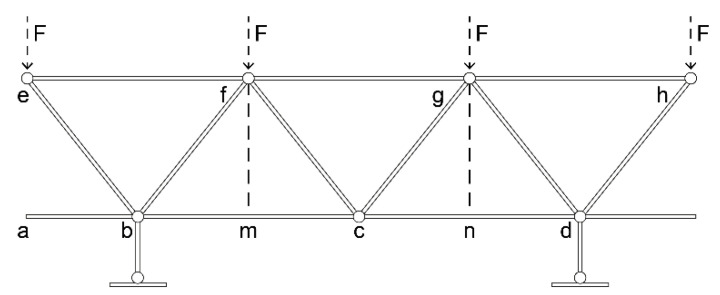
Force analysis of the Warren plane truss.

Section 2.1.1 discussed the instability of the resin reinforcement structure with square, double-sided shallow grooves, and cylindrical straight holes, and its tendency to brittle fracture owing to the bending moment generated by an external force. The resin-reinforcement structure with the Warren truss slot consists of a parallel chord truss structure with a symmetrically inclined rod support that is corrugated transversely and longitudinally, as shown in Figure 7.

From Figure 7, ∵ ab = bm = mc = cn = nd and eb = fb = fc = gc. Thus, ∴ ∠eba =∠fbm and ∠fcm = ∠gcn. Rods eb and fb, bf and cf, fc and gc, and cg and dg symmetrically support nodes b, f, c, and g, respectively. Regardless of the direction of external force F to the top chord, the truss structure was stable. Symmetrical diagonal web rods were axially stressed, thereby forming a two-force rod system.

#### 3.2.3. Three-Dimensional Tetrahedral Truss Lattice Cell Slot-Hole Resin Stiffener Structure

**Figure 8:**
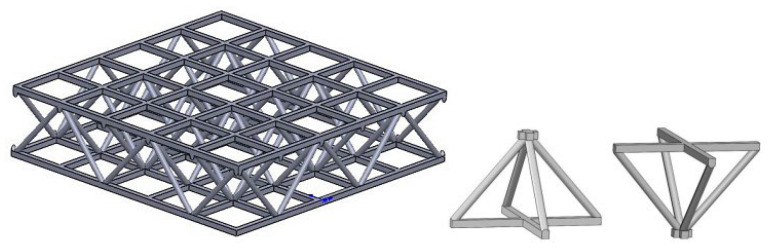
Warren truss slot resin reinforcement structure and the tetrahedral truss lattice cell.

In the VARI molding process, the resin infiltrated the corrugated symmetrically inclined holes in the transverse and longitudinal directions, and was cured to form a reinforcement structure. The core of the Warren truss slot resin reinforcement structure is a 3D tetrahedral truss lattice cell with an obverse and reverse staggering arrangement, as illustrated in Figure 8.

**Figure 9:**
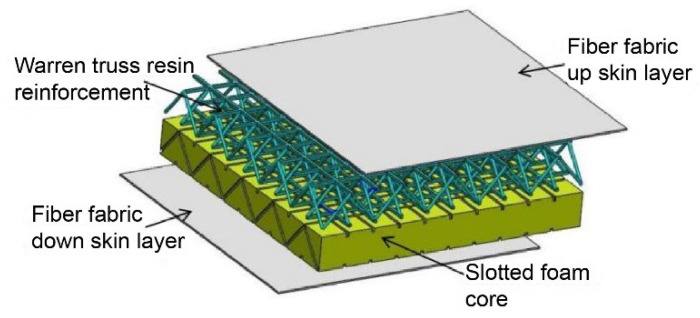
Diagram of the lattice cell Warren truss slot resin-reinforced foam sandwich structure.

The lattice cell in the core layer can effectively improve the strength and stiffness of the sandwich composites. Figure 9 shows a sandwich structure with a resin-reinforced Warren truss lattice cell as the core layer. The composite sandwich material consisted of a 3D lattice cell Warren truss resin reinforcement, and foam effectively integrated a 3D lattice cell truss structure with additive manufacturing, which strengthened the material, reduced its weight, increased functionality, and increased its suitability for large-scale manufacturing.

### 3.3. Bearing Capacity Simulation Analysis of Slot-Hole Resin-Reinforced Stiffener Structure 

#### 3.3.1. Mechanical Characterization of Cured Unsaturated Polyester Resin 

**Figure 10:**
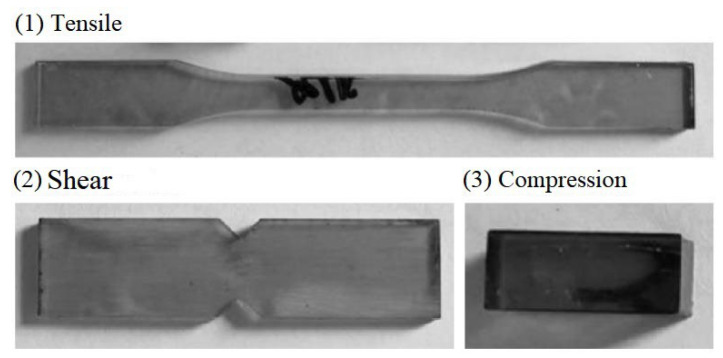
Test samples of cured unsaturated polyester resin.

Unsaturated polyester resin (Ashland Inc, Ashland, KY, USA. 105-60RT) and an M50 curing agent were selected for the experiment. Resin casting was performed by uniformly mixing the resin and curing agent at a ratio of 1.2% at temperatures in the range of 18–25 °C. The test sample was obtained after laser cutting and grinding according to the American Society for Testing and Materials (ASTM) D3039M [25], ASTM D5379 [26], and ASTM D695-10 [27] standards. The tensile sample characteristics were as follows: size = 200 mm × 20 mm × 3.5 mm, middle width = 20 mm, shear sample size = 60 mm × 20 mm × 3.5 mm, middle width of the V-groove on both sides = 25 mm, and the compression sample size = 25 mm × 10 mm × 10 mm. Photographs of the test samples are shown in Figure 10. The performance parameters of the cured resin, such as the tensile, compression, and shear, were tested and analyzed; the results are listed in Table 3. 

Each group contained five samples, and the average values of the test results were used as the performance parameter values; additionally, the standard deviations of the tensile strength, modulus, and Poisson’s ratio were relatively small, and the standard deviations of the tensile failure strain, compression strength, shear strength, and modulus were relatively large.

#### 3.3.2. Material Constitutive Model and Failure Criteria

**Figure 11:**
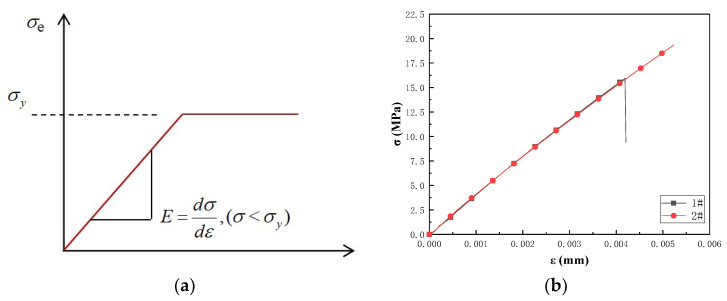
The verification of material model constitutive equation. (**a**) Constitutive equation curves of the material model. (**b**) Stress–strain curves of samples 1# and 2#.

**Figure 12:**
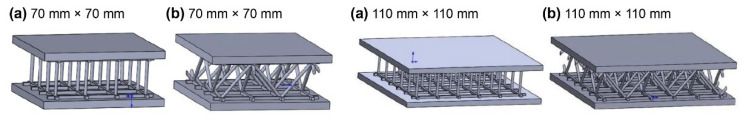
Flat compression simulation models. the double-sided, square shallow groove, and cylindrical deep holes (**a**) and optimized Warren truss with symmetrically inclined holes (**b**) resin stiffener structures.

The slot-hole resin stiffener structure of cured, homogeneous, unsaturated polyester resin was subjected to static compression and bending. The material of unsaturated polyester resin was assumed to undergo elastic deformation according to the isotropic linear elasticity theory. Additionally, the MAT_PLASTIC_KINEMATIC is the ANSYS LS-DYNA material model, and it was used to model the constitutive equation in this study; this model has the form

(6) $$E=\frac{d\sigma }{d\varepsilon },(\sigma <\sigma_{y})$$

where ε is the equivalent elasticity strain, and $\sigma_{y}$ is the yield stress at the non-zero elasticity strain. The constitutive equation curves of this material model are shown in Figure 11. The tensile stress–strain curve of cured 105-60RT unsaturated polyester resin of the samples 1# and 2# in Table 3 is shown in Figure 12. As shown, the stress–strain curves of samples 1 # and 2 # coincide with the constitutive equation curves of the material model.

Furthermore, the principal stress failure was used as the failure criterion. Table 4 lists the mechanical parameters of unsaturated polyester resin.

#### 3.3.3. Modeling and Condition Setting

The finite-element software ANSYS LS-DYNA package was used for the flat compression simulations, and the material properties of the cured unsaturated polyester were set according to Table 1. Flat compression simulations of the square, double-sided lattice slot with 88° inclined cylindrical holes (No. a) and the optimized Warren truss with symmetrically inclined holes (No. b) were performed. For each simulated resin reinforcement structure, the dimensions were set to 70 mm × 70 mm and 110 mm × 110 mm for a total of four models. The 3D modeling software SolidWorks was used to construct these 25 mm-thick compression simulation models, as shown in Figure 12. The resin stiffener structure was divided into a 2.5 mm tetrahedral mesh. A 5 mm hexahedral mesh was created for the compression plates at the top and bottom parts. The total number of mesh units ranged from 9000 to 40,000. The lower plate was subjected to a displacement load of zero in the X, Y, and Z directions. For the top plate, a displacement load of zero in the X and Y directions and −2.5 mm in the Z direction was applied. The computation time was set to 0.001 s.

**Figure 13:**
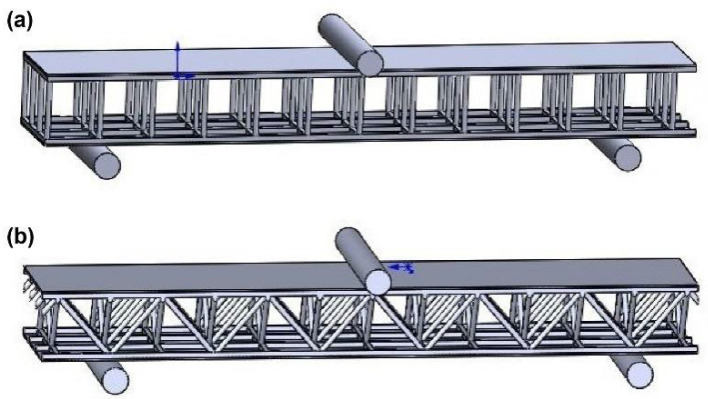
Bending simulation models. the double-sided, square shallow groove, and cylindrical deep holes (**a**) and optimized Warren truss with symmetrically inclined holes (**b**) resin stiffener structures.

The ANSYS LS-DYNA package was used for the bending simulations, and the material properties of the cured unsaturated polyester were set according to Table 1. The failure mode was set as the principal stress failure. A bending simulation of the double-sided, square lattice slot with a cylindrical straight hole (a) and an optimized Warren truss with symmetrically inclined holes (b) was performed. The size of the reinforcement structure was set to 250 mm × 62 mm, a thickness of 25 mm, and a span of 200 mm; the loading-rod diameter is 10, as shown in Figure 13. The bending model of the resin reinforcement structure was divided into a 2.5 mm tetrahedral mesh. A 3 mm hexahedral mesh was created for the top and bottom skin layers. The total number of mesh units was approximately 50,000. The bottom roller bars were subjected to a displacement load of zero in the X, Y, and Z directions. For the top roller bar, a displacement load of zero in the X- and Y-directions and −15 mm in the Z-direction was applied. The computation time was set to 0.002 s.

#### 3.3.4. Bearing Capacity from the Finite Element Simulation of Slot-Hole Resin-Reinforced Stiffener Structure 

**Figure 14:**
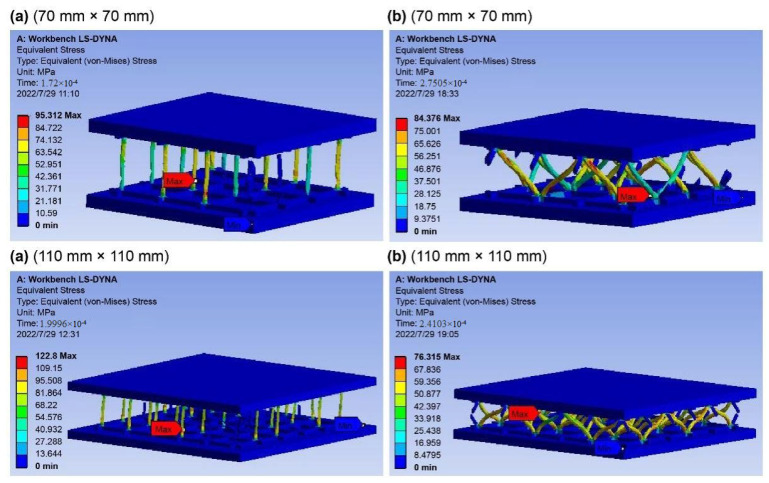
Flat compression failure stress distributions at the maximum external force. the double-sided, square shallow groove, and cylindrical deep holes (**a**) and optimized Warren truss with symmetrically inclined holes (**b**) resin stiffener structures.

The stress distribution p at the maximum external force of the double-sided, square shallow groove, and cylindrical deep hole (a) and the slotted Warren truss (b) resin reinforcement structure after the compression test simulations are shown in Figure 13. The failure nodal stress at the maximum external force of the slotted Warren truss (b) resin reinforcement structure is shown in Figure 14. The failure nodal stress at the maximum external force of the slotted Warren truss (b) resin reinforcement structure is smaller than that of the double-sided, square shallow groove, and cylindrical deep hole (a) resin reinforcement structures with a longer failure time. In addition, the resin-reinforced webs of the slotted Warren truss were bent after compression.

**Figure 15:**
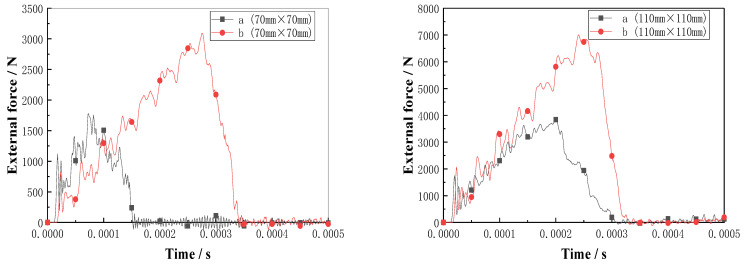
Plots of external force vs. time for the flat compression of the slot resin rib structures.

The maximum compressive loads of the double-sided, square shallow groove, and cylindrical deep hole (a) and slotted Warren truss (b) resin stiffener structures are plotted as a function of the failure time in Figure 15. As shown, the slotted Warren truss (b) resin stiffener structure, which supports symmetrical diagonal resin web stiffener, exhibits a better compressive bearing capacity that is twice that of the double-sided, square shallow groove, and cylindrical deep hole (a) resin stiffener structure with an extended failure time that was $5\times 10^{-5}$ s longer. 

**Figure 16:**
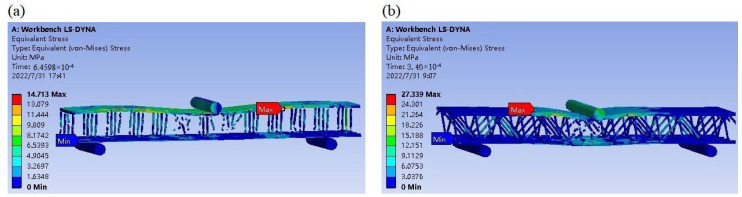
Bending failure stress nephogram at the maximum contact force. the double-sided, square shallow groove, and cylindrical deep holes (**a**) and optimized Warren truss with symmetrically inclined holes (**b**) resin stiffener structures.

The stress distribution plots of the bent slotted resin-reinforced structure at the maximum external force are shown in Figure 16. The failure node stress at the point with the maximum bending contact force of the slotted Warren truss (b) resin stiffener structure is greater than that of the double-sided, square shallow groove, and cylindrical deep hole (a) resin stiffener structure. The majority of the symmetrically inclined resin-reinforced web rods in the slotted Warren truss (b) resin stiffener structure remained intact at the maximum bending contact impact, whereas the vertical webs in the double-sided, square shallow groove, and cylindrical deep hole (a) resin stiffener structures were mostly broken. 

**Figure 17:**
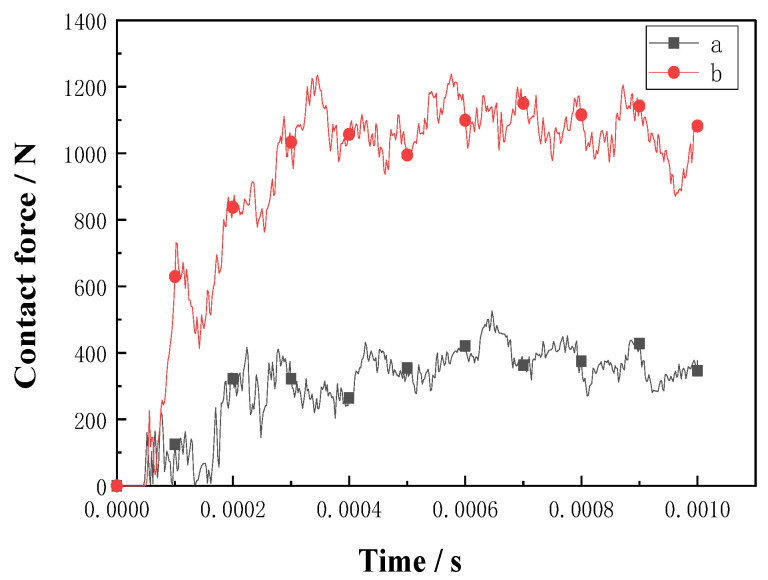
Plots of contact force vs. time for the bending of slot resin rib structure.

For the slotted holes (a) and (b) in the resin-reinforced structures, the maximum bending load was plotted against the failure time, as shown in Figure 17. The bending bearing contact force of the slotted Warren truss (b) resin stiffener structure was approximately 1.5 times higher than that of the double-sided, square shallow groove, and cylindrical deep hole (a) resin stiffener structure.

## 4. Experimental Characterization of Performance Improvement of the Optimized Slotted-Resin Stiffener-Reinforced Sandwich Composites

### 4.1. Materials and Methods

#### 4.1.1. Preparation Diagram of the Warren Truss Slot-Hole Foam-Core Plate

**Figure 18:**
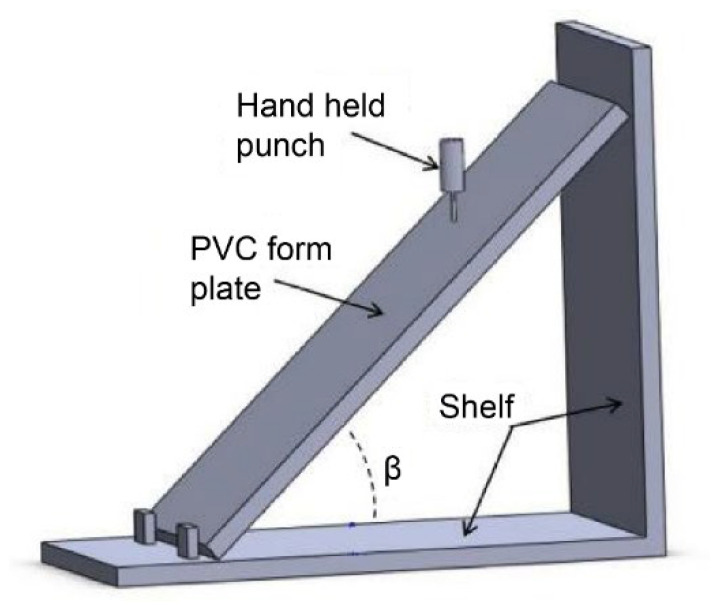
Preparation diagram of the Warren truss slot-hole foam-core plate.

Using a 25 mm thick PVC rigid foam board as the original material, conventional cutting was first employed to process the square shallow grooves on the foam board surface with 20 mm transverse and longitudinal groove spacings. As the current XYZ triaxial drilling method cannot achieve inclined hole drilling, a custom inclined foam plate holder was fabricated. Based on the thickness of the foam board, and its longitudinal and transverse groove spacings, the inclined hole was calculated to be β = arctan(2025) = 48.65°, which was also the tilt angle of the foam board. An electric drill with a ∅2 mm drill bit was used to drill vertically at the crossing center of the longitudinal and transverse surface shallow grooves, as shown in Figure 18. A foam-core board containing a slotted Warren truss structure with double-sided, square shallow grooves, and corrugated symmetrical inclined holes was prepared.

#### 4.1.2. Flat Compression and Bending Test Samples Preparation

**Figure 19:**
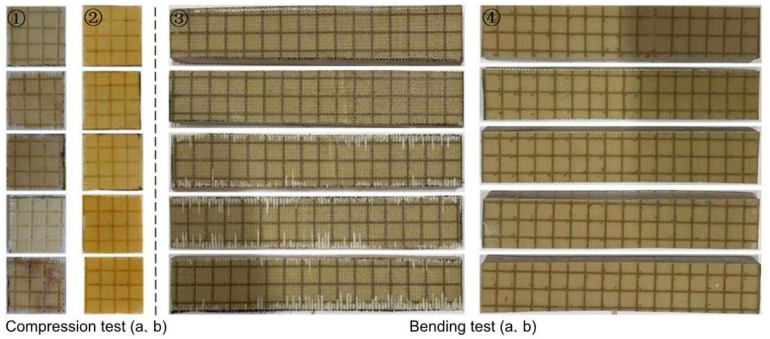
Five samples of flat compression and bending test, 1—compression test samples (**a**), 2—compression test samples (**b**), 3—bending test samples (**a**), and 4—bending test samples (**b**).

A DIAB H60 PVC rigid foam core with a density of 60 kg/m^3^ was used as the foam core. The unsaturated polyester resin (Ashland Inc. 105-60RT) and M50 curing agent were mixed at a ratio of 1.2%. The resin-reinforced slotted Warren truss (b) sandwich composite and the resin-reinforced sandwich composite with double-sided square lattice grooves and cylindrical straight holes (a) were manufactured using the VARI process. The flat compression test sample was cut to a size of 76.2 mm × 76.2 mm, according to the ASTM C364-16 [28] standard, and the bending test sample was cut to a size of 300 mm × 60 mm, according to the ASTM C393-16 [29] standard. The test samples are shown in Figure 19.

#### 4.1.3. Flat Compression and Bending Test Method

**Figure 20:**
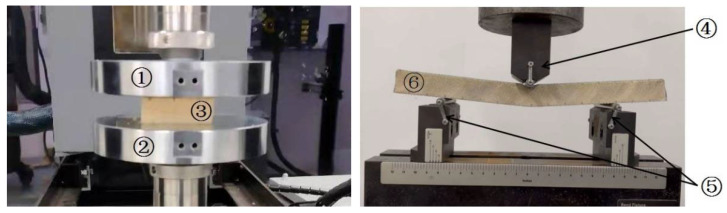
Flat compression and bending test, 1—upper flat pressing fixture, 2—down flat pressing fixture, 3—flat compression test sample, 4—upper loading-rod, 5—down braces, 6—bending test sample.

An MTS Landmark 370.25 electronic universal tester was used for the evaluation. For each sandwich composite type, five qualified test samples were selected for the flat compression tests. Figure 20 shows photographs of the flat compression test method. Compression was applied at a load rate of 1 mm/min until the structure failed. The compressive strength of the structure was calculated as,

(7) $$\sigma_{t}=\frac{P}{A},$$

where *σ_t_* is the compressive strength, *P* is the applied load, and *A* is the cross-sectional area of the specimen. 

A landmark testing system was used for the three-point bending test. Five test samples were tested for each sandwich composite type, as shown in Figure 20. Bending was applied at a load rate of 1 mm/min until the sample was crushed. The flexural strength of the structure was obtained as follows:

(8) $$\sigma_{f}=\frac{p\times l}{4b\times t_{f}\times \left(h–t_{f}\right)},$$

where *σ_f_* is the flexural strength, *p* is the crosshead load, *b* is the specimen width, *h* is the specimen thickness, *l* is the span, and *t_f_* is the surface-layer thickness.

### 4.2. Experimental Results and Comparative Analysis of the Resin-Reinforced Foam-Core Sandwich Composite Bearing Capacity 

#### 4.2.1. Experimental Results and Comparative Analysis for Flat Compression Test

**Figure 21:**
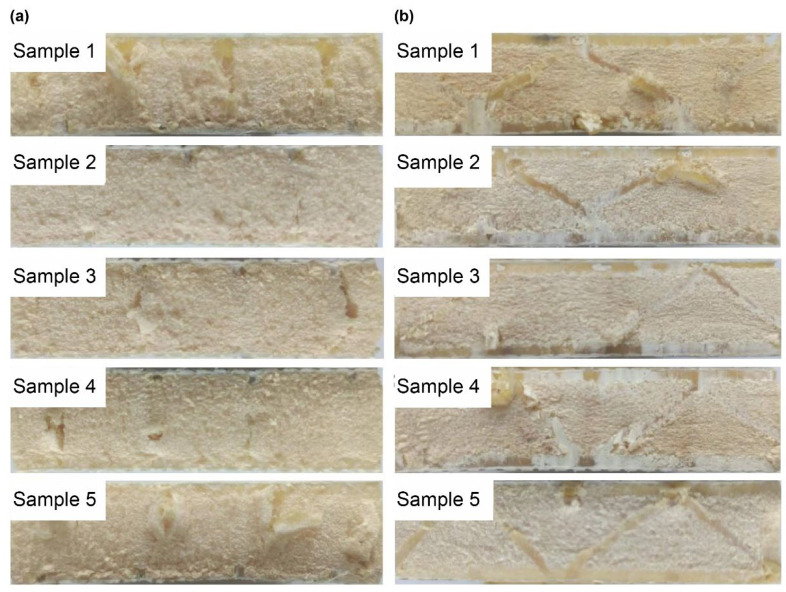
Side images demonstrating crack failure of the flat compression samples (**a**) and samples (**b**) after the flat compression test.

**Figure 22:**
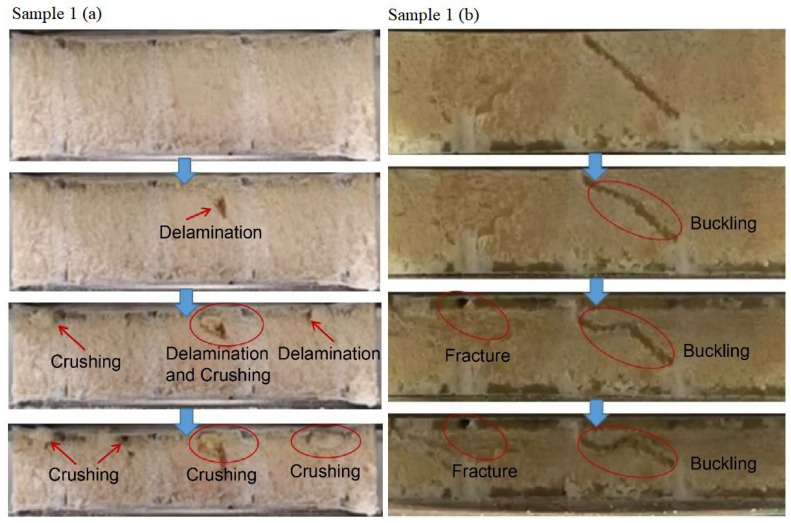
Side images demonstrating crack failure process of sample 1 (**a**) and sample 1 (**b**) after the flat compression test.

The flat compression and bending simulation analysis of the slot-hole resin stiffener structure in Section 3.1 demonstrated that optimizing the design of the slot-hole body considerably increased the bearing capacity of the slot resin stiffener structure after the design of the slot-hole body was optimized. The two sets of slotted resin-reinforced foam-core sandwich composite samples failed (lateral ruptures) after flat compression testing. Photographs of the ruptures are shown in Figure 21. When resin-reinforced foam-core sandwich composites with double-sided, square lattice shallow grooves, and cylindrical straight holes (a) were pressed, the straight cylindrical resin stiffener was fractured, and the resin-reinforced foam was also ruptured and partially dislodged. Local buckling and deformation of the symmetrically inclined resin structure occurred when the slotted Warren truss (b) resin-reinforced sandwich composites were stressed. Moreover, local delamination in the resin reinforcement structure and foam core was observed. The photographs of the flat compression tests for sample 1 of the slotted-hole-type (a) and (b) sandwich composites are shown in Figure 22.

As shown in Figure 22, sample 1 of the resin-reinforced foam sandwich composite with double-sided, square shallow grooves, and cylindrical straight holes (a) was subjected to compression, and the delamination of the slotted resin-reinforced foam occurred followed by crushing. When sample 1 of the Warren truss-slotted (b) resin-reinforced sandwich composite was compressed, the symmetrically inclined resin reinforcement structure was bent, and buckling and fracture occurred.

**Figure 23:**
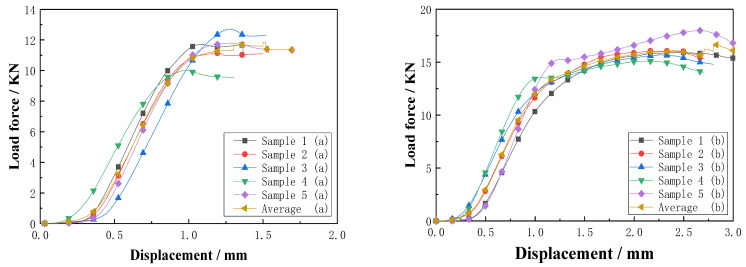
Load–displacement curves of the slot resin-reinforced sandwich composites (a) and (b) by flat compression test.

The compression test load of the slotted-hole type (a) and (b) resin-reinforced sandwich composite specimens are plotted as a function of displacement in Figure 23. For the resin-reinforced sandwich composite with double-sided, square shallow grooves, and cylindrical straight holes, the characteristic curves were similar; however, data dispersion exists. The load reached a maximum value in the range 10.02–11.70 kN at a displacement of 1.25 mm. Dispersion was observed in the load–displacement curves of the Warren truss-slotted resin-reinforced sandwich composite, although the slopes of the curves were approximately the same for each specimen. The load reached a maximum value in the range 15.11–17.99 kN at a displacement of 2.5 mm. The displacement of the Warren truss-slotted resin-reinforced sandwich composite specimen subjected to the maximum load was approximately twice that of the sandwich composite with double-sided, square shallow grooves, and straight cylindrical holes. The maximum load capacity and corresponding compressive strength of the two slotted-hole-type foam-core sandwich composites are listed in Table 5. 

Each group included five samples, and the average values of the test results were used as the performance parameter values. The calculations of the data in Table 5 show that the standard deviations of the maximum flat compression load and flat compression strength of the two sets of slot-hole, resin-reinforced, sandwich-composite samples were all relatively small; the average maximum load capacity of the resin-reinforced sandwich composite with double-sided square shallow grooves and straight cylindrical holes was 11.77 kN with a dispersion of 8.6%. The average compressive strength was 1.96 MPa with a dispersion of 5.5%. In the case of the Warren truss-slotted resin-reinforced sandwich composite, the average maximum load capacity was 16.16 kN with a dispersion of 6.7%, and the compressive strength was found to be 2.74 MPa with a dispersion of 7.9%. Compared with the slot structure with double-sided square shallow grooves and cylindrical straight holes, the load capacity of the Warren truss slot resin-reinforced foam-core sandwich composite increased by 4.7 kN, and the compressive strength performance improved by approximately 40%. 

#### 4.2.2. Experimental Results and Comparative Analysis for Bending Test

**Figure 24:**
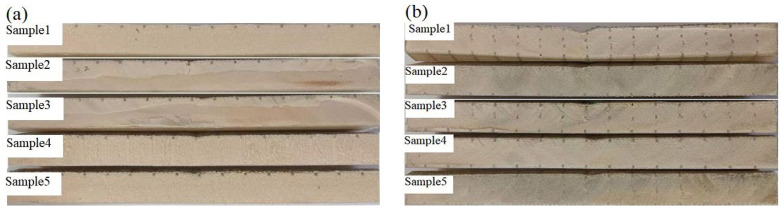
Side images of the crack failures of samples (**a**) and (**b**) during the bending test.

The images of the Warren truss-slotted, and square, double-sided shallow grooves, cylindrical straight-hole, and resin-reinforced sandwich composite are shown in Figure 24. During the three-point bending test, local delamination and debonding occurred in the middle sections of the specimen impact areas, and the Warren truss-slotted resin-reinforced sandwich composite was optimized. The sandwich composite specimens exhibited good resilience and maintained excellent integrity and straightness after bending. 

**Figure 25:**
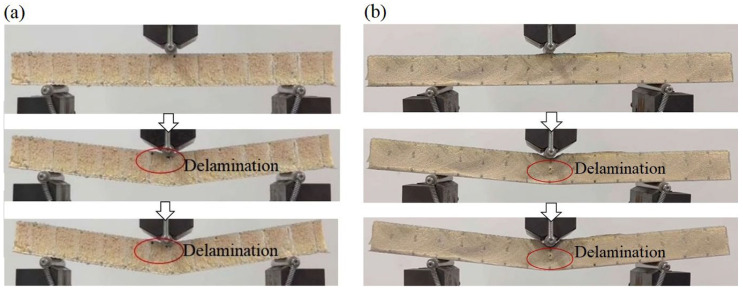
Side images of the crack failure process of samples 3 (**a**) and sample 3 (**b**) of the two types of slotted-hole foam-core sandwich composites by bending test.

The bending test failure process for sample 3 of the slotted-hole, foam-core sandwich composites (a) and (b) is depicted in Figure 25. When sample 3 was subjected to the three-point bending, delamination occurred at the interface of the resin-reinforcement structure and foam. During the bending test of the Warren truss-slotted foam-core sandwich composite, delamination occurred on the symmetrically inclined resin-reinforcement structure and foam with the gradual bending of the specimen.

**Figure 26:**
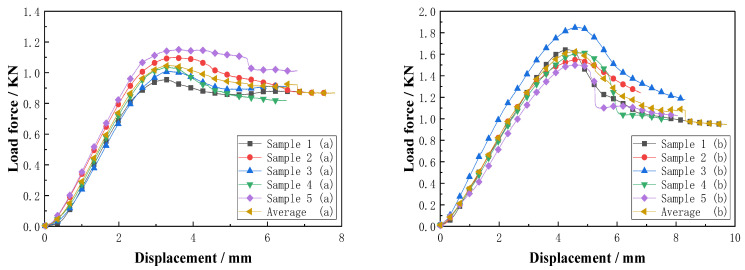
Load–displacement curves of the slotted resin-reinforced sandwich composites (a) and (b) during the bending test.

The load–displacement relationship of the resin-reinforced foam-core sandwich composites with two types of slotted-hole structures is illustrated in Figure 26. For the resin-reinforced foam-core sandwich composite with double-sided, square shallow grooves, and cylindrical straight holes, the curves are similar with minimal variations. The maximum bending load was 0.96–1.15 kN. For the Warren truss-slotted resin-reinforced foam-core sandwich composite, data dispersion was observed with a similar slope for each curve. The maximum bending load was 1.50–1.85 kN. Compared with the double-sided, square shallow grooves, and cylindrical straight holes, the displacement of the Warren truss-slotted resin-reinforced foam-core sandwich composite subjected to the maximum bending load was 50% higher. The maximum bending load and corresponding bending strength of the slotted-hole types (a) and (b) resin-reinforced sandwich composites are summarized in Table 6.

As indicated earlier, each group contained five samples, and the average values of the test results were used as the performance parameter values. The calculations of the data in Table 6 shows that the standard deviation of the maximum flat bending load and bending strength of the two sets of slot-hole, resin-reinforced, sandwich-composite samples were relatively small. The average maximum bending load of the resin-reinforced foam-core sandwich composite with square, double-sided shallow grooves, and cylindrical straight holes was 1.05 kN, with a dispersion of 7.2%. The average bending strength was 45.58 MPa with a dispersion of 6.7%. In the case of the Warren truss-slotted resin-reinforced sandwich-foam-core composite, the average maximum bending load was 1.63 kN and the dispersion was 8.2%. The average bending strength was 74.58 MPa and the dispersion was 8.5%. Compared with the square, double-sided, shallow groove with cylindrical, straight hole, resin-reinforced sandwich composite material, the maximum bending load of the slotted Warren truss, resin-reinforced, sandwich-foam-core composite increased by 0.58 kN, and the bending strength performance improved by approximately 60%.

A comparison of the experimental results suggests that the failure mode and load-bearing capacity of resin-reinforced sandwich composites with different slot-hole structures of the foam core can vary considerably. For the resin-reinforced sandwich composite with squared shallow surface grooves and cylindrical straight holes, the delamination of the resin and foam occurred under pressure, followed by the early rupture of the straight resin stiffener web. For the optimized Warren truss slot resin-reinforced foam-core sandwich composite, delamination of the resin and foam core occurred when the material was stressed, accompanied by the bending and fracture of the symmetrically inclined webs. Moreover, higher load displacements were obtained. Compared with the resin-reinforced foam sandwich composite with double-sided square shallow grooves and cylindrical straight holes, the maximum compression and bending capacity of the Warren truss-slotted resin reinforcement foam-core sandwich composite improved by more than 50%. The compressive and bending strengths also substantially improved by 40% and 60%, respectively, indicating an enhanced overall loading capacity.

## 5. Conclusions

In this study, a slot structure with double-sided, square shallow grooves and cylindrical straight holes was optimized for a Warren truss-slotted structure. The Warren truss structure consisted of double-sided square shallow grooves and longitudinally and transversely corrugated, symmetrically inclined holes. The core was composed of tetrahedral truss lattice cells with obverse and reverse staggering. The following conclusions were drawn from this study:

1. Processing the foam-core slot-hole structure with semiautomatic triaxial cutting and drilling can lead to instability because the cylindrical straight holes may not be completely perpendicular to the squared shallow grooves on the surface. When the resin reinforcement structure was stressed, the web was subjected to a specific bending moment and became susceptible to brittle fracture, this significantly reduced the overall bearing capacity of the slotted resin stiffener structure with double-sided square shallow grooves and cylindrical straight holes, and its reinforcement effect on the sandwich composite material.
2. The inclined support rods were essential to the stability and bearing capacity of the truss, and the lattice cell can improve the specific stiffness, specific strength, and toughness of the sandwich structure. In this study, the double-sided, square shallow grooves, and cylindrical straight hole structure was optimized and improved to a Warren space truss slot-hole structure. This Warren truss structure was composed of double-sided square shallow grooves and longitudinal and transverse corrugated symmetry through inclined holes, and the middle core was a tetrahedral truss lattice cell element with a staggered positive and negative arrangement.
3. During the VARI molding process, the amount of resin used to infiltrate the Warren truss slot structure was similar to that of the structure with square shallow grooves and cylindrical straight holes. However, the simulated compression load capacity of the optimized Warren truss-slotted resin stiffener structure doubled, and its maximum bending load was 1.5 times higher. When subjected to stress, the symmetrically inclined resin-reinforced web rods were first bent, followed by fracture failure. The failure occurred at a later time than that of the resin stiffener structure with double-sided square shallow grooves and straight cylindrical holes.
4. Compared to the double-sided, square shallow grooves, and cylindrical straight hole resin-reinforced foam-core sandwich composite, the flat compression load capacity of the optimized Warren truss-slotted resin-reinforced foam-core sandwich composite increased by 4.7 kN, and the compressive strength increased by approximately 40%. The bending load increased by 0.58 kN, and the bending strength improved by approximately 60%, demonstrating superior bearing capacity performance.

To maintain the conventional parameters, this study used a standard 25-mm-thick foam board as the original material. The square shallow groove sizes on both sides were kept constant, and the straight cylindrical holes were optimized into Warren truss slots with symmetrically inclined holes. 

The foam-core slot structure was usually processed using semiautomatic triaxial cutting and drilling, resulting in an unstable resin-reinforced stiffener structure after the fluid resin was injected and cured in the slot holes; additionally, the resin-reinforced web was susceptible to brittle fracture that affected the overall load-bearing capacity of this resin-reinforced stiffener structure. Therefore, this study ensured the stability of an inclined strut-truss structure and the strong bearing capacity of lattice cells, and optimized the poor slot-hole structure into a Warren space truss slot-hole structure, wherein the central core structure comprised tetrahedral truss lattice cells. The compression load capacity and structural stability of the optimized Warren truss-slotted, resin-reinforced stiffener structure were greatly improved, as indicated by simulation analyses. As revealed by the experimental test outcomes, the maximum compression and bending capacity and strengths of the Warren truss-slotted, resin-reinforced foam-core sandwich composite were greatly improved, and the amount of resin used to infiltrate the Warren truss slot structure was not increased. The study findings are expected to be significant for the design of foam-core plate slot-hole structures, for the improvement in the strength of form-core sandwich composite materials, and for the widening of the range of applications of these materials in the field.

## Figures and Tables

**Table 1:** Weight proportions in the groove resin structure of the sandwich composite.

Foam-Core Sandwich Composite Test Panel (1 m × 1 m, Weight = 5.94 kg)				
Structural Materials	Glass Fiber Fabric	Foam Core	Resin	Slot Resin Stiffener Structure
Weight (kg)	1.60	1.48	1.95	0.91
Percentage (%)	26.9	25.0	32.8	15.3

**Table 2:** Unit parameter values of Warren truss.

*p*/mm	*w*/mm	*d*/mm	*Φ*/mm	*s*/mm	*h*/mm
20	2	2	2	20	25

**Table 3:** Performance parameters of the cured 105-60RT unsaturated polyester resin.

Mechanical Property	Tensile				Compression		Shear	
	Strength/MPa	Modulus/GPa	Failure Strain	Poisson’s Ratio	Strength/MPa	Modulus/GPa	Strength MPa	Modulus/GPa
1#	15.9	3.59	4274.00	0.40	18.50	1.46	11.10	1.13
2#	19.3	3.47	5490.00	0.39	19.10	1.50	15.70	1.88
3#	20.9	3.05	6824.00	0.34	17.70	1.22	12.50	1.51
4#	18.7	3.23	5628.00	0.38	31.70	1.36	13.9	1.62
5#	15.9	3.14	4709.00	0.37	22.20	1.65	12.1	1.43
average (AVG)	18.14	3.30	5385.00	0.37	21.84	1.44	13.10	1.51
standard deviation (STDEV)	1.97	0.20	875.45	0.02	5.16	0.14	1.60	0.25

**Table 4:** The unsaturated polyester resin elastic parameters.

Property	Value
density (kg/m^3^)	1500
isotropic elasticity	-
Young’s modulus (GPa)	3.3
Poisson’s ratio	0.4
bulk modulus (GPa)	5.5
shear modulus (GPa)	1.1786
principal stress failure	-
maximum tensile stress (MPa)	2.5
maximum Shear Stress (MPa)	1.15

**Table 5:** Flat compression test results of the slot resin-reinforced sandwich composite core test samples.

Performances	Double-Sided Square Shallow Groove Cylindrical Straight Hole							Warren Truss Slot						
	1#	2#	3#	4#	5#	AVG	STDEV	1#	2#	3#	4#	5#	AVG	STDEV
Maximum flat compression load/kN	11.70	11.16	12.71	10.02	11.77	11.47	0.88	15.93	16.09	15.69	15.11	17.99	16.16	0.97
Flat compression strength/MPa	2.01	1.98	2.02	1.76	1.96	1.95	0.095	2.69	2.65	2.74	2.52	3.10	2.74	0.19

**Table 6:** Bending test results of the slotted resin-reinforced sandwich composite core test samples.

Performances	Double-Sided Square Shallow Groove Cylindrical Straight Hole							Warren Truss Slot						
	1#	2#	3#	4#	5#	AVG	STDEV	1#	2#	3#	4#	5#	AVG	STDEV
Maximum bending load/kN	0.96	1.10	1.01	1.03	1.15	1.05	0.07	1.64	1.55	1.85	1.62	1.50	1.63	0.12
Bending strength/MPa	41.76	48.88	44.82	43.95	48.50	45.58	2.73	76.84	71.35	83.71	74.28	66.73	74.58	5.67

## Data Availability

All data are available in the article.
